# The Impact of Stalking and Its Predictors: Characterizing the Needs of Stalking Victims

**DOI:** 10.1177/08862605231185303

**Published:** 2023-07-24

**Authors:** Jennifer E. Storey, Afroditi Pina, Cherise S. Williams

**Affiliations:** 1 University of Kent, Canterbury, UK; 2 Royal Holloway University of London, Egham, Surrey, UK

**Keywords:** consequences, victimization, negative outcomes, persistent harassment, national

## Abstract

Victims of stalking suffer severe and varied impacts requiring assessment and treatment. Research to inform support is limited. This study examines a national sample of stalking victims to identify the types and prevalence of impact reported and the predictors of impact. A secondary analysis of 258 stalking cases reported to a stalking charity was conducted. Four categories of victim reported impact were coded; psychological and substance abuse, physical health, practical impact on life, and impact on others. Stalking duration, severity, the diversity of stalking behaviors, and the relationship between the victim and perpetrator were investigated as predictors of impact. In all, 48 types of impact were identified with victims experiencing an average of four types. Psychological impact was the most prevalent (91.5%). Several new forms of impact were identified including a variety of impacts on persons known to the victim (e.g., children, friends) in 35.3% of the sample. Increased diversity of stalking behavior was predictive of impact in all models (explaining 11% of the variance in total impact scores), except for physical impact which was not analyzed due to low prevalence. Stalking impact was prevalent and varied, suggesting that victims (and potentially those close to them) require trauma-informed support from clinicians. Future research should include the development of a stalking impact index to improve the consistency of research and clinical assessment of need.

Stalking is a crime with varied and severe psychological, physical, and practical impacts on victims from repeated and unwanted contact by perpetrators that cause victims to experience fear for their safety or the safety of others (Matos et al., 2019). The mental health and well-being impact of stalking make victims a population of clinical interest due to requirements for the assessment and treatment of their needs. Lifetime prevalence rates of stalking in Western populations range from 2% to 15% (Whyte et al., 2011). Similarly, the Crime Survey for England and Wales (2019) found that adults aged 16 to 74 reported lifetime prevalence rates of 6.5%, with 1.5% of respondents having been stalked in the last year.

Stalking is an intrusive and damaging crime. To maximize assistance and support for victims, the specific needs of victims stemming from the impact of stalking victimization should be targeted. These varied needs will most often require the assistance of mental health professionals, but also health and social care and support professionals, to assess/diagnose, treat, and refer victims to appropriate services. The Treatment Improvement Protocol (TIP) “endorses a trauma-informed model of care; this model emphasizes the need for behavioral health practitioners and organizations to recognize the prevalence and pervasive impact of trauma on the lives of the people they serve and develop trauma-sensitive or trauma-responsive services” (Substance Abuse and Mental Health Services Administration, 2014). To engage in this care model, professionals require guidelines, based on a robust research literature, on the breadth and nature of impact experienced from the trauma. The aim of this study is to contribute to that literature by examining a national sample of stalking victims to identify the types and prevalence of impact reported and the stalking characteristics that predict impact.

## Victim Impact Studies

Studies on the victim impact of stalking have generally taken two forms, studies of impact types and prevalence and studies of predictors of impact. Several studies have focused on stalking impact, reporting types, and prevalence of impact (e.g., Acquadro Maran & Varetto, 2018; Amar, 2006; Dressing et al., 2005, 2014; Kamphuis & Emmelkamp, 2001; Logan et al., 2006; Pathé & Mullen, 1997; Stieger et al., 2008; Westrup et al., 1999). Participants who were the victims of stalking were gathered from criminal justice, clinical, undergraduate, and community samples. Across studies, there is a great deal of overlap in the types of impact identified. The impact types can be broadly classified as follows: (i) psychological impact and substance abuse, (ii) health impact, (iii) practical impact on life and activities, and (iv) impact on third parties.

### Psychological Impact and Substance Abuse

The psychological impacts of staking were examined and identified most frequently across studies. Fear of death or physical harm was reported by 43% to 97% of victims (Amar, 2006; Kamphuis & Emmelkamp, 2001; Morgan & Truman, 2022). Anxiety was reported by 44% to 88% of victims (Dressing et al., 2005; Pathé & Mullen, 1997; Stieger et al., 2008), with 12% to 14% experiencing panic attacks (Dressing et al., 2005; Stieger et al., 2008) and 55% experiencing flashbacks and intrusive thoughts (Pathé & Mullen, 1997). Between 26% and 34.6% of victims reported depression (Dressing et al., 2005, 2014; Stieger et al., 2008) and 24% considered or attempted suicide (Pathé & Mullen, 1997). Thirty-seven percent of victims met the criteria for a diagnosis of posttraumatic stress disorder (PTSD; Pathé & Mullen, 1997). The authors suggest that this may be linked to violence experienced in the relationship which was also related to PTS symptoms. Three quarters of victims (75%) felt powerless (Pathé & Mullen, 1997) and over half (55.4%) felt helpless (Dressing et al., 2014).

Aggressive thoughts were experienced by 31% to 65% of victims (Dressing et al., 2005, 2014; Stieger et al., 2008; Pathé & Mullen, 1997) and 56% to 61% felt agitated (Dressing et al., 2005; Stieger et al., 2008). Increased suspicion was experienced by 39% to 44% of victims (Dressing et al., 2005; Stieger et al., 2008) and victims reported mistrust toward others (68.2%; Dressing et al., 2014) and reticence toward unknown people (49.6%) (Dressing et al., 2014). Victims reported feeling afraid to enter new relationship 32.6% (Dressing et al., 2014) and social withdrawal 34.6% to 38% (Dressing et al., 2014; Pathé & Mullen, 1997). Concentration problems 48.1% (Dressing et al., 2014), loss of control 34.6% (Dressing et al., 2014), and feelings of inner unrest 78.2% (Dressing et al., 2014) were also common. A clinically significant level of psychomedical symptoms were experienced by 59% of victims (Kamphuis & Emmelkamp, 2001). Substance use was reported by 23% of victims (Pathé & Mullen, 1997).

Rather than offer overall prevalence rates, some studies compared victims of stalking to other groups (e.g., non-victims, victims of other types of violence) or by gender. Compared to non-victims, victims of stalking have significantly lower WHO-5 Well-Being Index scores than non-victims (e.g., 52%–57% of victims vs. 27% of non-victims scoring in the pathological range), more symptoms of PTSD and score higher on subscales of the Symptom Checklist-90-R, specifically obsessive compulsive, interpersonal sensitivity, and depression (Dressing et al., 2005, 2014; Stieger et al., 2008; Westrup et al., 1999). Furthermore, Amar (2006) found that victims, compared to non-victims, reported more somatization, depression, and hostility as well as significantly higher levels of general psychological distress.

Logan et al. (2006) compared victims of moderate intimate partner violence to victims of severe intimate partner violence, and victims of severe intimate partner violence including stalking. Results showed that victims of stalking (compared to intimate partner violence alone) showed more PTSD, depression, and anxiety. Significantly fewer of the stalking victims reported *no* mental health problems (9%) compared to victims of moderate (25%) and severe (34%) intimate partner violence.

Acquadro Maran and Varetto (2018) compared female and male stalking victims. Physical symptoms and emotional symptoms were identified as impacts of stalking. Except for anger, which was significantly more common among males, all symptoms were reported more often among females but not to a significant degree.

### Physical Health

Sleep disturbances are widely reported, impacting between 30% and 74% of victims (Dressing et al., 2005, 2014; Pathé & Mullen, 1997; Stieger et al., 2008). Just over half of victims (55%) surveyed by Pathé and Mullen (1997) reported feeling excessive tiredness or weakness. Appetite disturbances (48%) (Pathé & Mullen, 1997) and weight fluctuations (45%) were also reported (Pathé & Mullen, 1997) as were stomach and bowel issues (19%–44.1%) (Dressing et al., 2005; Dressing et al., 2014; Stieger et al., 2008; Pathé & Mullen, 1997). Between 14% and 47% of victims reported experiencing headaches (Dressing et al., 2005, 2014; Stieger et al., 2008; Pathé & Mullen, 1997). Sick leave was taken by 18% of victims in one study (Dressing et al., 2005). Finally, Amar (2006) found that victims of stalking, compared to non-victims, were more likely to report poorer physical health status.

### Practical Impact on Life and Activities

The types and prevalence of practical impacts on victims’ lives and activities are wide ranging. Between 20% and 82% of victims report changing their lifestyle to avoid the stalker (Amar, 2006; Dressing et al., 2005; Kamphuis & Emmelkamp, 2001; Pathé & Mullen, 1997; Stieger et al., 2008). Additional security was employed by 17% to 73% of victims (Dressing et al., 2005; Kamphuis & Emmelkamp, 2001; Pathé & Mullen, 1997; Stieger et al., 2008). Amar (2006) found that 38% of victims took extra precautions. Limiting social activities or leaving the house was reported by 9% to 70% of victims (Amar, 2006; Kamphuis & Emmelkamp, 2001; Pathé & Mullen, 1997). A decrease in or cessation of work or school attendance was reported by 4% to 53% of victims (Amar, 2006; Dressing et al., 2005; Kamphuis & Emmelkamp, 2001; Pathé & Mullen, 1997), changes in workplace, school, or career were reported by 2% to 37% (Amar, 2006; Dressing et al., 2005; Pathé & Mullen, 1997; Stieger et al., 2008). Between 2% and 39% of victims moved to a new home, city, or state (Amar, 2006; Dressing et al., 2005; Kamphuis & Emmelkamp, 2001; Pathé & Mullen, 1997; Stieger et al 2008). Changing phone numbers and installing an answerphone were actions taken by 13% to 62% of victims (Amar, 2006; Dressing et al., 2005; Kamphuis & Emmelkamp, 2001; Stieger et al., 2008). Six percent of victims wore concealing clothes or accessories and 1% changed vehicles (Amar, 2006), 7% to 69% of victims sought legal counsel (Dressing et al., 2005; Kamphuis & Emmelkamp, 2001; Pathé & Mullen, 1997; Stieger et al., 2008), 12% to 44% consulted medical practitioners (Dressing et al., 2005; Pathé & Mullen, 1997; Stieger et al., 2008), and 1% hired a private investigator (Amar, 2006).

### Impact on Others

The impact on individuals other than the victim has rarely been examined, only Dressing et al. (2014) reported on such an impact, identifying “partnership problems” in 22.3% of cases. However, “partnership problems” was not defined and thus the extent to which the victim’s partner was impacted cannot be ascertained. The sample included cases of cyberstalking only.

## Predictors of Impact

The second type of study that has been conducted on the impact of stalking examines what victim and situational characteristics predict victim impact. The severity of stalking behavior was identified as a predictor of victim impact in two studies (Kamphuis et al., 2003; Mechanic et al., 2000). Mechanic et al. (2000) found that among battered women who experienced stalking (*N* = 66), severe types of stalking (i.e., relentless as opposed to infrequent stalking) predicted increased impact and was associated with more severe psychological and physical outcomes such as increased rates of distress, depression, and PTSD as well as more abuse, violence, and injuries. Similarly, when Kamphuis et al. (2003) examined PTS in 131 victims of stalking by a prior intimate partner, stalking severity (including duration, variation, and violence) accounted for 22% of the PTS variance with violence being the strongest predictor. Personality (lower openness) and coping style (passive) were also associated with increased PTS, explaining 8% of the variance.

The relationship between the victim and perpetrator and the diversity (or variety) of stalking behavior have also been identified as predictive of stalking impact across multiple studies. Sheridan and Lyndon (2012) examined the hypothesis that prior victim–perpetrator relationship would be more predictive of stalking consequences (physical, psychological, social, and economic) than gender in a sample of 1,214 victims. The hypothesis was only supported in the case of social consequences. Relationship was predictive of physical, psychological, and social consequences, whereas female gender was predictive of physical and psychological consequences.

Johnson and Kercher (2009) investigated predictors of negative outcomes of stalking defined as negative psychological consequences. Victims (*N* = 123) were classified based on level of impact, where group one had a high probability of experiencing almost all the negative psychological consequences of victimization queried, group two was classified as moderate and was more likely to report a lack of concentration and wanting to be alone but had fewer serious consequences compared to group one, and group three was low and unlikely to report negative consequences aside from anger. Those victims receiving government assistance were significantly less likely to be in the low impact group suggesting that they experienced heightened impact from stalking victimization. Increased impact of stalking was associated with a previous relationship between the victim and the perpetrator, an increased number of stalking behaviors experienced, an increased variety of stalking behaviors and longer duration of stalking.

Finally, Matos et al. (2019) surveyed a community sample of stalking victims (*n* = 236) about the presence of seven categories of impact: professional or academic performance, physical health, psychological health, relationships with others, intimate relationships, economics/finances, and lifestyle, on a four-point Likert scale. Category items were not provided. They found impact was predicted by diversity, frequency, and fear of stalking behavior, explaining 38% of the variance in their model.

The studies conducted to date on the impact of stalking provide a great deal of insight into the consequences of stalking for victims as well as some of the factors that might predict impact severity. The majority of the studies in this area are at least a decade old; thus, updated literature is needed given significant changes to stalking methods due to technological advances and changes to legal practices. Studies to date also typically used predetermined lists of impacts and asked victims to indicate which they had experienced (e.g., in the form of health questionnaires). Although helpful in identifying symptoms, these can limit spontaneous self-report, thereby limiting the variety of impacts identified by victims to only those anticipated by the researchers. Moreover, most frontline workers do not have additional time to spend on lengthy psychological questionnaires and must assess the problem that is reported to them and provide assistance based on the information available. This is particularly true of charities that rely on volunteer support and who struggle to meet the high demand for their services. This is salient in the United Kingdom (UK) which has multiple charities dedicated to supporting victims of stalking, both at the national (e.g., *the Suzy Lamplugh Trust, Paladin*) and local (e.g., *Protection Against Stalking* in Kent, *Veritas* in Sussex) levels. The Suzy Lamplugh Trust (SLT) is the largest national stalking charity, supporting the widest range of victims in the UK and runs The National Stalking Helpline (NSH) for victims of stalking. Thus, examining the information available to frontline workers is critical to understanding the type of information that is reported by victims and what information about impact can be identified and assessed from those reports.

## Current Study

To gain this knowledge, the present study examines the spontaneous self-reported impact of stalking on victims and the characteristics of the stalking that predict this impact using a national sample gathered from victims seeking help from the SLT. In comparing rates of impact to previous studies, the results identify the extent to which victims spontaneously self-disclose impacts such as depression and indicate whether more probing questions by report takers and clinicians are needed. Furthermore, by examining help-seeking victims, the results can directly inform clinical practice by identifying the support that victims deem necessary.

Two research questions are examined. First, what are the types and prevalence of impacts spontaneously reported by help-seeking victims of stalking? Second, what factors predict stalking impact? Based on the previous literature (Johnson & Kercher, 2009; Kamphuis et al., 2003; Matos et al., 2019; Mechanic et al., 2000; Sheridan & Lyndon, 2012), we hypothesize that the following factors will predict increased stalking impact: diversity of stalking behavior (i.e., more types), increased severity of stalking behavior, increased duration of stalking, and a closer relationship between the victim and perpetrator.

## Method

### Overview

Information on victim impact and potential predictor variables was gathered from 258 reports of stalking made to The National Stalking Helpline (NSH). Information from cases reported to the NSH by phone or email was recorded electronically in client records by trained volunteers or employees (hereafter referred to as helpline advisors). Client records included the impact of stalking as reported by the caller. Access to records was provided by the SLT and the project received ethical approval. The study was preregistered on The Open Science Framework (https://osf.io/hnwbs); however, data were not included to protect victim privacy. Client records were coded using a coding sheet and anonymized prior to analysis. Grouping of the stalking behaviors and data analysis followed the format used in Kamphuis et al. (2003). The coding of impact was led by the data and the grouping of impact types led by the prior research as reviewed above. The interrater reliability for the behavior coding and the impact coding were examined and found to be excellent.

### Cases

The SLT runs the NSH which responds to phone calls and emails from targets of stalking. For each reported instance of stalking, helpline advisors compile an electronic client record. Repeated callers are linked via the identifiable information they provide (e.g., name, date of birth) as well as a unique service ID. The service ID was used herein to link cases with the same victim for coding and ensure each included victim was unique. The first 271 client records from the NSH in 2018 were examined for inclusion in the study. Four records were excluded because the circumstances described did not meet the legal definition of stalking in the UK. A further nine cases were excluded because a review of the records suggested that the reporters of stalking were experiencing delusions due to impaired psychological health rather than stalking. For instance, they reported that people were watching them through the TV or that red beams were following them. Exclusion of these types of reports was a practice suggested and performed by the NSH. Thus, a total of 258 unique client records were included in the study. For clarity, the targets of stalking in those records will be referred to as *victims* of stalking, the individual(s) engaging in stalking behavior will be referred to as *perpetrators* of stalking and the overarching incident reported will be referred to as the *case*.

Information was coded from client records by two researchers who were also trained as volunteers at the SLT. Client records are populated by helpline staff based on information obtained from victims. Staff undergo training to understand the record system, what information needs to be recorded and how to record that information reliably. For instance, staff select a duration of stalking from a fixed list of time periods. New staff have their work reviewed until it is determined that they can work independently. Client records included demographic information, a list of the stalking behaviors experienced by the victims, the duration of the stalking, the relationship held between the victim and the perpetrator, and a free-text section of case notes. The free-text section of the client record was available in all cases and was where helpline advisors would record spontaneous details of stalking impact and sometimes stalking behaviors. Email inquiries were copied directly into the free-text section. Information from phone calls were collated and summarized in this section based on a set assessment template. Only senior members of staff gathered information by phone. Free-text sections included half to one and a half pages of text. Client records also included reports from external agencies like police and independent domestic violence advisors which were also coded for impact information.

### Materials

The information available for this study was that ordinarily collected in the context of a report of stalking to the NSH and retained in client records. No additional scales or questions were added as this best reflects the information that frontline staff have time to collect and have available to them. Client records were coded using a coding sheet to extract demographic information (gender, age), predictor variables (i.e., perpetrator victim relationship, duration of stalking, diversity of stalking behaviors, and severity of stalking), and the outcome variable (i.e., impact).

#### Predictor Variables

##### Perpetrator–Victim Relationship

Perpetrator victim relationship was classified in decreasing level of closeness as ex-intimate (which included only significant relationships such as ex-wife/husband, ex-boyfriend/girlfriend), family, acquaintance (e.g., friend, coworker, short-term dating relationship), and stranger.

##### Duration of Stalking

The duration of the stalking was recorded by the NSH as less than a month, 1 to under 3 months, 3 to under 6 months, 6 to under 12 months, 1 to under 2 years, 2 to 5 years, 5 to 10 years, over 10 years, and over 20 years. Numerical values were assigned to this ordinal scale to create a continuous scale for analysis.

##### Diversity of Stalking Behaviors

The diversity of stalking behaviors was measured by the number of different types of stalking behavior that the perpetrator engaged in. Thus, a more diverse case would be one where multiple behaviors were used (e.g., texts, following, hacking, and threats) rather than fewer behaviors (e.g., only emails) regardless of the frequency of those behaviors. Stalking behaviors were defined by the NSH (see Table 1). For example, watching was when perpetrators were visibly watching the victim, whereas spying was when perpetrators took steps to discreetly monitor the victim such as through windows or using cameras. The electronic records system included tick boxes for helpline advisors to indicate which of 26 behavior types were present in each case. For the purposes of this study, 25 behavior types were retained and coded as present or absent in each case. The one excluded behavior type was “In through work” which represented stalking behaviors that occurred at work, such as sending an email to work, visiting work. These behaviors were also captured under other behavior types such as email and visit and thus the “In through work” type was excluded to avoid double counting stalking behaviors. On occasion, stalking behaviors were detailed in the free-text field and not ticked off on the list of 25 behavior types. When this occurred, the behaviors were added to the existing list of types of behavior by the coder (AP). To ensure accuracy, a second coder (CSW) independently rated the presence of the behaviors and interrater reliability was calculated. Reliability was indexed using Cohen’s kappa coefficient (κ) for the categorical ratings of the two raters (AP and CSW), scores ranged from .85 to perfect agreement (1.00), which is considered almost perfect agreement (Cohen, 1960). Total scores for the number of behavior types experienced were calculated from the 25 presence ratings for each rater. Reliability was indexed using intraclass correlation coefficients (ICC_1_; two-way mixed effects model, absolute agreement method). ICC_1_ for behavior total scores was of .95, which is considered excellent (Fleiss, 1986).

**Table 1.** Prevalence of Stalking Behavior Types Spontaneously Reported by Victims.

**Category**	**Stalking Behavior**	***n***	**%**
**Unwelcome communication**		**220**	**85.3**
	Texts	130	50.4
	Calls	130	50.4
	Social Media	104	40.3
	Emails	85	32.9
	Letters	48	18.6
	Gifts	41	15.9
**Contact**		**192**	**74.4**
	Visiting home/work	98	38.0
	Following	75	29.1
	Harassing	74	28.7
	Loitering	70	27.1
	Watching	61	23.6
	Hacking	30	11.6
	Spying	24	9.3
	Breaking and entering	19	7.4
	Tracking	8	3.1
	Monitoring	6	2.3
**Associated behaviors**		**139**	**53.9**
	Third party contact	104	40.3
	Vexatious complaints	49	19.0
	Criminal damage	46	17.8
**Violent stalking behaviors**		**127**	**49.2**
	Threats	103	39.9
	Death threat	34	13.2
	Physical assault	24	9.3
	Suicide threat	16	6.2
	Sexual assault	8	3.1
	Revenge porn	5	1.9

*N* = 58.

The stalking behavior types available for analysis were those developed by the NSH. To increase comparability with prior research, the three impact studies that ran predictive analyses were examined to determine whether their methods and results could be mapped on to the available data. Kamphuis et al. (2003) identified the most sophisticated classification system (similar to a previous study by Mullen et al., 1999) that also fit the content of the NSH data. For example, the categories from Johnson and Kercher (2009) could not be fit to the data because the NSH behaviors were recorded without any specification regarding their nature (e.g., emails were not further classified as angry or apologetic). Kamphuis et al.’s (2003) classification system was also preferable as it ranked behavior severity. Thus, the 25 behaviors recorded by the NSH were grouped into the four categories specified by Kamphuis et al. (2003, p. 150): (i) unwelcome communication including “telephone calls, mail, email and graffiti,” (ii) contact including “following, maintaining surveillance and approaching the victim,” (iii) associated behaviors including “involving third parties such as the victim’s children, giving or ordering on the victim’s behalf, damaging property, and initiating spurious legal actions,” and (iv) “violent stalking behaviors including threats and actual assault.” The grouping of the NSH behaviors in the four categories is shown Table 1.

##### Severity of Stalking

The four categories specified by Kamphuis et al. (2003) were also used to classify the severity of the stalking behavior. Category four, violent stalking behaviors, was considered the highest severity followed by category three, associated behaviors; category two, contact; and category one, unwelcome communication which was the lowest severity. The highest severity category experienced by each victim was recorded to reflect the level of stalking severity. For instance, if a victim experienced stalking behaviors from categories one and four, they were classified as category four, the highest severity level.

#### Outcome Variable

##### Impact

The impact of stalking was recorded in and coded from the free-text section of the NSH client records. All mentions of impact in the free-text section were recorded as written into a narrative list for each case. The lists of impacts for each case were then reviewed for commonalities (e.g., mentions of depression, needing medication) and categories of impact were developed (CSW) based on those commonalities to facilitate coding (i.e., Depression psychological impact, Psychological medication, Medication for physical impact). A second coder then reviewed and discussed the categories (JES). Cases were then coded using the categories, where each impact category was marked as present (1) or absent (0) (AP). Next, a second rater (JES), blind to the first ratings, coded a sample of the cases. Discrepancies in coding were discussed and the coding tool was refined further. This process was then repeated and there was complete agreement in the coding of the second sample. Reliability testing, using ICC_1_ as above, was then completed on a random sample of 15% (*n* = 39) of cases, with two raters (JES and AP) blind to each other’s ratings, ICC_1_ was .97 indicating excellent agreement.

For analysis, the impact of stalking reported was organized in two ways. First, a total impact score was created by summing the number of present impacts in each case. This score identified the total number of impacts that the victim described to the NSH. An impact total score was selected as a method of analysis because it would indicate the extent to which total impact in a case is influenced by the predictor variables. Second, based on the research literature reviewed in the introduction and the categories of impact identified in the sample, four overarching impact groupings were identified: (i) psychological impact and substance abuse (i.e., impaired psychological well-being), (ii) physical health impact (i.e., impaired physical health), (iii) practical impact on life and activities (i.e., changes to day-to-day life), and (iv) impact on others (i.e., impact on individuals other than the victim). The impacts experienced in each grouping were summed to provide a total score for each group. Predictors of impact were examined by impact grouping since it provides a more nuanced indication of the influence of various predictors on the impact of stalking. Where differences in impact vary by predictor, this could be used by professionals in practice to pre-emptively direct victims to services that could reduce a specified impact type. The impact categories and four impact groupings are displayed in Table 2.

**Table 2.** Prevalence of Impact and Comparison to Ranges in Previous Studies.

Impact Category	Impact Type	*n* (%)	Prevalence (%) Range From Prior Impact Studies
Psychological and substance abuse		236 (91.5)	
	Fear	178 (69)	43%–97%^a,e,f^
	Distress	66 (25.6)	—
	Secondary victimization	42 (16.3)	—
	Vulnerability	40 (15.5)	—
	Fear for life	36 (14)	—
	Hypervigilance	33 (12.8)	—
	Anxiety	31 (12)	44%–88%^c,g,h^
	Helplessness	28 (10.9)	55.4%^d^
	Conflicting feelings	26 (10.1)	—
	Depression	15 (5.8)	26%–34.6%^c,d,h^
	Shame or embarrassment	14 (5.4)	—
	Difficulty managing emotions	14 (5.4)	—
	Panic attacks	11 (4.3)	12%–19.7%^b,c,h^ (latter value is from Amar (2006) and classed as a physical symptom)
	Guilt	11 (4.3)	—
	Suspicious distrust	10 (3.9)	39%–68.2%^c,d,h^
	Anger	8 (3.1)	50.3%^a^
	Posttraumatic stress disorder	4 (1.6)	37%^g^
	Nightmares	4 (1.6)	—
	Suicidal ideation	4 (1.6)	24%^g^
	Substance misuse	1 (.4)	23%^g^
Physical health		35 (13.6)	
	Insomnia	12 (4.7)	30%–74% (sleep disturbance)^c,d,g,h^
	Medication	10 (3.9)	—
	Physical health	9 (3.5)	(poorer physical health status)^b^
	Long-term sick	6 (2.3)	18% (sick leave)^c^
Practical impacts on life and activities		140 (54.3)	
	Isolation	53 (20.5)	9%–70%^a,c,e,g^
	Disable social media and/or email	32 (12.4)	—
	Move home	27 (10.5)	2%–39%^b,c,e,g,h^
	Installation of home security	20 (7.8)	17%–73% (additional security)^c,e,g,h^
	Other employment problems caused	20 (7.8)	—
	Change phone number	20 (7.8)	13%–62%^b,c,e,h^
	Other financial impact	16 (6.2)	—
	Change travel routes	15 (5.8)	20%–82% (changing lifestyle)^b,c,e,g,h^
	Creation of safety plan	14 (5.4)	—
	Use of bodycam and/or panic alarm	9 (3.5)	17%–73% (additional security)^c,e,g,h^
	Quit employment	8 (3.1)	4%–53% (decrease in or cessation of work or school attendance)^b,c,e,g^
	Counseling related to practical impact	7 (2.7)	—
	Victim investigated for stalking due to spurious accusation by stalker	7 (2.7)	—
	Victim investigated by social services due to spurious accusation by stalker	6 (2.3)	—
	Employment transfer	5 (1.9%)	2%–37% (changes to workplace, school, or career)^b,c,g,h^
	Homelessness	2 (0.8)	—
	Court-related fees	4 (1.6)	7%–69% (sought legal counsel)^c,e,g,h^, 1% private investigator^b^
	Had to pay for expert assistance	4 (1.6)	
Impact on others		91 (35.3)	
	Children	37 (14.3)	—
	Family	28 (10.9)	—
	Friends	22 (8.5)	—
	Partner	18 (7)	22.3%^d^
	Neighbors	6 (2.3)	—
	Colleagues	8 (3.1)	—

a Acquadro Maran and Varetto (2018), ^b^Amar (2006), ^c^Dressing et al. (2005), ^d^Dressing et al. (2014), ^e^Kamphuis and Emmelkamp (2001), ^f^Morgan and Truman (2022), ^g^Pathé and Mullen (1997), ^h^Stieger et al. (2008).

*N* = 258.

### Procedure

Permission to use the data was obtained from the SLT and ethical approval for the study was obtained from the lead researcher’s university. In addition, a data sharing agreement was also in place between the researchers and the SLT. Access was provided to electronic client records where the client had given consent for their information to be used for research or evaluation purposes.

The first 271 cases taken on by the NSH in 2018 were examined for inclusion in the study. The 258 cases that met the inclusion criteria were coded using a coding sheet to extract the information identified above. Initial coding of the database was completed by CSW, who was a volunteer for NSH at the time and who initially used the data as part of an unpublished MSc dissertation. Prior to engaging with cases, CSW underwent screening by the SLT including a Disclosure and Barring Service check (i.e., a criminal record check). She was then trained as a member of staff. This initial coding of the database was done to anonymize the data and collect only that which was relevant to the study prior to removing the data from the SLT. This study utilized a subset of that data and employed different coding and analysis procedures to the unpublished dissertation.

### Data Analyses

Analyses were conducted using SPSS v 27, IBM. Descriptive statistics including frequency analysis were used to report demographic characteristics, information about the predictor variables (diversity of stalking behavior, severity of stalking, duration of stalking, and relationship between the victim and perpetrator) and impact; the latter also answers the first research question.

To examine research question two, the results, where possible, replicate the method used in Kamphuis et al. (2003). First, intercorrelations between the predictor variables were calculated using Pearson correlations. Second, stepwise regression analyses were employed with impact as the dependent variable. Prior to running the regression, we ran both a power analysis and an assumption check. Using G*power (version 3.1.9.6 for Mac OS X), we ran a sensitivity power analysis as we were constrained in our sample size. The effect (Cohen’s *f*^2^) detectable in our stepwise regression with a power of .95 and a significance level of .05 with a sample size of 258 and four predictors is 0.073. Thus, we concluded that we had the power to detect a small effect in our sample. Next, we evaluated whether the predictors in the models were multicollinear by estimating a variance inflation factor (VIF) for each predictor. The largest VIF value observed was 1.40. We therefore concluded that multicollinearity was not an issue. Finally, the normality of the residuals was examined using a QQ plot. The residuals followed the plotted straight line indicating that the assumption was not violated.

Following these checks, we ran the stepwise regression. First, the total impact score (i.e., the sum of impacts experienced by the victim) was included as the predictor variable, then the sum of each of the four impact groupings (i.e., psychological and substance abuse, physical health, practical impacts on life and activities, and impact on others) was examined separately to determine if predictors differed by impact type.

## Results

### Demographics

Stalking victims were 39 years of age on average (*SD* = 10.93) with a range of 17 to 82 years (age was missing in 33 cases). Most victims were female (*n* = 201, 77.9%, information was missing in three cases) and most perpetrators were male (*n* = 180, 69.8%, information was missing in 17 cases).

### Predictor Variables

#### Stalking Duration

Stalking duration varied from under 1 month to over 20 years, with about half of the sample experiencing stalking for 12 months or less (49%), the modal duration was 1 to under 2 years (*n* = 42, 16.3%).

#### Relationship type

The most common victim–perpetrator relationship was ex-intimate (*n* = 143, 55.4%), followed by acquaintance (*n* = 80, 31%), stranger (*n* = 16, 6.2%), and family (*n* = 11, 4.3%), relationship was unknown in 8 (3.1%) cases.

#### Diversity and Severity of Stalking Behavior

The prevalence of each type of behavior experienced by the stalking victims is found in Table 1 along with the prevalence of each of the four categories identified by Kamphuis et al. (2003). Each case included between 1 and 18 stalking behaviors with a median of five behaviors (*M* = 5.62; *SD* = 3.17) per case. Unwelcome communication was the most common behavior experienced by victims (85.3%). Both contact (74.4%) and associated behaviors (53.9%) were also common and experienced by more than half of victims. Violent stalking behavior, the most severe category, was experienced by almost half of the victims (*n* = 127, 49.2%). The most severe behaviors experienced by the remaining victims were associated behaviors (*n* = 60, 23.3%), followed by contact behaviors (*n* = 45, 17.4%) and unwelcome communication (*n* = 26, 10.1%).

### Intercorrelations Between Predictor Variables

Table 3 displays intercorrelations between the four predictor variables. Low to moderate correlations were identified. Diversity and severity of behavior shared the largest correlation.

**Table 3.** Intercorrelations Between Predictor Variables.

Predictor variables	1	2	3	4
1. Diversity of stalking behavior		.51*	.13	.29*
2. Severity of stalking behavior			−.03	.25*
3. Length of stalking behavior				−.05
4. Relationship between target and stalker				

*Note*. Relationship is coded from least close (stranger) to closest (intimate partner).

* *p* < .01.

### Dependent Variable

#### Impact

A total of 48 different types of impacts were identified by victims, the prevalence of each is identified in Table 2 along with the prevalence of the four impact categories. For comparison, Table 2 also includes the prevalence of the impact types identified in the studies reviewed in the introduction. Our results show that on average, victims reported four types of impact (*M* = 3.88, *SD* = 2.69) that the stalking had on them, with a range of no impact (*n* = 6) to 16 types of impact (*n* = 1). Psychological impact was most prevalent, followed by practical impacts on life and activities, impacts to others known to the victim, and finally physical impacts.

### Regression Analysis

Table 4 shows the results of the stepwise regression analysis where relationship type as well as diversity, severity and length of the stalking behavior were examined as predictors of impact total scores, as well as of the four impact types. With the exception of physical impact which was not calculated due to low prevalence, increased diversity of stalking behavior emerged as a significant predictor of impact across all models: total *R*^2^ = .11, *F*(1, 211) = 25.93, *p* < .001; psychological *R*^2^ = .09, *F*(1, 211) = 21.63, *p* < .001; practical *R*^2^ = .03, *F*(1, 211) = 7.07, *p* = .006; and impact on others *R*^2^ = .03, *F*(1, 211) = 7.12, *p* = .002. Diversity explained 11% of the variance in total impact scores, 9% for psychological, 3% for practical, and 5% for impact on others. Victim–perpetrator relationship was also a significant predictor of impact on others, where increased relationship closeness was inversely related to impact on others *R*^2^ = .05, *F*(2, 211) = 6.18, *p* = .025.

**Table 4.** Summary of Stepwise Regression Analysis Predicting Impact Total and Impact Types from Stalking Diversity, Severity, Length, and Victim–Perpetrator Relationship.

**Variable**	***B***	***SE B***	β	***t***	***p***
**Total impact**					
Constant	2.40	.36			
Diversity	.27	.05	.33	5.09	**<.001**
Severity			.14	1.85	.066
Length			−.03	−.47	.636
Relationship			.02	.28	.781
**Psychological impact**					
Constant	1.40	.22			
Diversity	.15	.03	.31	4.65	**<.001**
Severity			.10	1.34	.182
Length			−.06	−.86	.394
Relationship			−.02	−.22	.825
**Practical impact**					
Constant	.64	.17			
Diversity	.07	.03	.19	2.78	.**006**
Severity			.12	1.58	.116
Length			.08	1.13	.259
Relationship			.08	1.10	.271
**Impact on others**					
Constant	.54	.16			
Diversity	.05	.02	.22	3.20	.**002**
Severity			.09	1.09	.277
Length			−.07	−1.01	.314
Relationship	−.11	.05	−.16	−2.26	.**025**

*Note*. Total impact Adj *R*^2^ = .11, psychological impact Adj *R*^2^ = .09, practical impact Adj *R*^2^ = .03, impact on others diversity Adj *R*^2^ = .03, relationship Adj *R*^2^ = .05.

The Bold values significance level Provides final column of (p).

## Discussion

The aims of this study were to identify the prevalence of stalking impact in a national sample of self-reporting victims and to identify the predictors of stalking impact. A wide range of impact types, some of which expand on those currently in the research literature, were identified. Impact from stalking was common, with few victims reporting no impact. Impact was predicted by increased diversity of stalking behavior experienced, and in the case of impact to others by reduced closeness in the victim–perpetrator relationship (i.e., impact was predicted by stranger rather than ex-intimate relationships), thereby only providing support for our hypothesis around diversity. The results confirm and expand on prior literature and have important clinical implications as they highlight a victimized population (with almost half experiencing behaviors classified in the most severe violent behavior category) in need of clinical assessment and care. Furthermore, they indicate prevalent and varied needs (e.g., 91.5% experienced varied psychological and substance abuse-related impacts like anxiety and depression suggesting a need for mental health assessment and treatment) that are predicted by the behavioral diversity of the stalking.

This study contributes to the limited but growing body of research on the impact of stalking. Several novel impacts of stalking were identified in each of the four impact categories. A wider variety of impact types were reported by victims in our study. Of note was the category, impact on others. Previously, only impact on partners had been reported in one study, but our results reveal that stalking impacted multiple other individuals such as children, family, friends, and colleagues. This finding is critical because it identifies a larger circle of individuals who can potentially be impacted by stalking. This means there may be more individuals in need of support services and suggests that to resolve the needs of a stalking victim, support for those known to the victim may be required. The relevance of this to clinical practice is discussed further in the clinical implications section.

Novel and serious types of impact were also identified across the other three impact categories including secondary victimization, being investigated due to vexatious complaints by the perpetrator, homelessness, and needing to take medication. Although many new types of impact were identified, potentially due to the method used (spontaneous report rather than a preexisting scale), Table 2 reveals that across studies there was lack of consistent terminology and that this may result in an overestimation of unique impact types. For instance, previous studies included impacts such as powerlessness, sadness, and apprehension, which were not identified in our study; however, we did find impacts labled as helplessness, depression, and fear. Furthermore, there was a lack of agreement across studies regarding which types of impact fit within each category. For instance, we classified panic attacks as a psychological impact while Amar (2006) classified them as physical. We chose to classify them as psychological because although panic attacks have distinct systemic physical manifestations, they are classified as a psychological disorder in the DSM-V.

Many of the types of impact identified in our study were less prevalent among victims compared to previous studies. In some instances, this may be due to the nature of the samples examined, where we reviewed a sample of victims seeking help from a charity and others have examined criminal justice samples. Nevertheless, this would not account for all comparisons across studies. Differences may also reflect the fact that we did not use a questionnaire to solicit responses and relied instead on spontaneous victim recall. Thus, while our approach increased the variety of impact types reported, it may have come at the cost of capturing prevalence. These facts and findings, coupled with the lack of consistency in language used across studies to identify impact types suggests the need for an overarching and comprehensive impact index, based on the research to date with room for “other” reported impact to reflect the everchanging nature of stalking behavior. The clinical benefits of such an index are discussed in the clinical implications section. For the purposes of research, such an index would help to establish reliable impact prevalence rates and improve comparisons across studies and different types of victim samples.

Intercorrelations showed that diversity and severity of behavior were most highly correlated. As might be expected, this suggests that as perpetrators engage in more types of behavior, some of these behaviors will be more severe (this could also represent a pattern of escalation in some cases over time). Increased behavior diversity and severity were also associated with closer relationships. It would be expected that with greater victim access (e.g., intimate partners will have more access to their victim’s home, work, computer, etc., than would a stranger), you would see more diverse stalking behaviors. Increased severity of stalking in closer relationships is very much in line with the previous literature, particularly on intimate partners and violence in stalking (Senkans et al., 2021).

The results also indicated that overall and across impact types, the diversity of stalking behaviors was predictive of impact. This partially supports our hypothesis and is an important finding because it indicates an avenue to identify victims who are vulnerable to high stalking impact. Our data show that it is not the severity of stalking or duration that predicts stalking impact (contrary to our hypothesis), rather the variety of stalking behaviors. One explanation for these results is that diverse stalking behaviors impact the victim’s life in multiple ways and when victims are impacted on multiple fronts, this may have a substantial cumulative effect on their well-being and/or may mean that they have fewer safe spaces where they can retreat to and recover from the impacts of the stalking intrusions. Often when assessing impact and the subsequent need for support, we naturally gravitate to the severity of the stalking behavior for guidance because it can be a sign of significant negative outcomes for victims (e.g., physical harm). The results suggest that when assessing need, we must also consider the diversity of stalking behaviors encountered by victims and prioritize highly diverse cases for support.

Contrary to our hypothesis, severity, duration, and victim–perpetrator relationship were not predictive of impact and in the case of the latter not in the expected direction given that relationship closeness was inversely related to impact on others. The inverse relationship could be the result of stranger perpetrators causing more fear than those known to the victim and those around them. Stranger perpetrators may cause more fear due to the unknown nature of their intentions or capabilities and this could result in greater impact due to the lack of perceived predictability. The lack of significance for duration might be explained by a limitation in the way in which the duration data were grouped in the dataset which did not allow an examination of duration via a continuous number of months.

The results must be considered in line with the limitations and strengths of this study. Due to the nature of the sample, we relied entirely on spontaneous victim reports. As noted above, not providing a list of impacts likely resulted in an underestimation of impact. Despite this, we chose to report on this sample due to its unique characteristics, as a national sample of help-seeking victims self-reporting impact, which we felt could add to the research literature. Due to its unique nature and the study design, we identified new impact types and are able to make novel recommendations such as the creation of an impact index to more completely capture victim impact. Furthermore, due to the help-seeking and self-reporting nature of the sample, the results are highly relevant to mental health professionals who will most often see victims who are seeking help based on self-identified need(s).

### Clinical Implications

Given the help-seeking nature of our sample and our findings, this study has several important clinical implications. Many of the stalking impact types identified require clinical assessment and diagnosis and the majority of impact types could benefit from mental health treatment. For instance, the most prevalent impact category was psychological and substance abuse which was present for 91.5% of victims and included anxiety, depression, difficulty managing emotions, panic attacks, anger, PTSD, and suicidal ideation. The heightened need for mental health care among victims who have experienced stalking supports the importance of trauma informed practice. Key elements of trauma-informed practice include knowledge of the prevalence of trauma, recognition of how trauma impacts all individuals involved and use of this knowledge in practice (Substance Abuse and Mental Health Services Administration, 2014).

In relation to these key elements, our findings revealed issues around identifying the prevalence and types of impact that victims experience. Impact was highly varied and underreported which we attribute in part to the spontaneous self-report nature of the data used in this study where victims may only report what they immediately consider as threatening and may leave out other types of impact that perhaps in their view are less salient or critical. Clinicians cannot be expected to be aware of all types of stalking impact, thus, as above, we suggest that an index of stalking impact be developed. The development of such an index could utilize the methodology of McEwan et al. (2021) who developed indices measuring stalking victimization (Stalking Assessment Indices Victimization or SAI-V) and perpetration (the Stalking Assessment Indices Perpetration or SAI-P). The structure of the SAI could also be utilized, where victims are asked a series of questions. The present findings and past research could be used as a starting point for the development and validation of such a tool in line with the methods of McEwan et al. (2021). Using the four broad categories of impact identified herein (i.e., psychological, physical, practical impacts, and impact on others) as well an “other impact” option could increase capture of novel impact.

The development of an impact index would enhance our existing provisions and equip clinicians and frontline responders with the necessary information to assist victims in fully identifying and reporting stalking and its impact as well as make educated decisions about risk and support provision. Furthermore, both researchers and practice professionals note the necessity of considering impact in the identification of stalking. In their SAI development paper, McEwan et al. (2021) state that it is “essential to combine conduct with some measure of victim impact or perpetrator intent when identifying a pattern of behavior as stalking” (p. 437). In the United Kingdom, impact is included in the legal definition of stalking (Section 4A (1)(b)(ii) of the Protection from Harassment Act, 1997 Offence-Stalking involving fear of violence or serious alarm or distress), where stalking is having a substantial adverse effect on a victim’s usual day-to-day activities. This addition to the Stalking Legislation from 2012 recognizes the overall emotional and psychological harm caused by stalking even where an explicit fear of violence is not created by each incident of stalking behavior. Therefore, an impact index could be beneficial to police officers investigating reports of stalking and harassment as it would help to evidence a charge of stalking. A list of impact types will also help victims to recognize impact that they were suffering from but had not previously linked to stalking (e.g., stomach or weight issues) and comprehensively recall those impacts that are pertinent to them i when reporting to police and clinicians. As noted, any such index should leave room for reporting other impact types. Due to technology, stalking is an ever-evolving crime and with time the impact of stalking will change, thus such an index will require review and updating. In addition to helping clinicians to comprehensively assess and treat victims’ needs, this index would also improve interprofessional communication and inform appropriate client referrals. In this way, all professionals involved with the victim could engage in trauma-informed practice and be aware of all pressing needs for support.

Results showed a greater variety of impact on those known to the victim than in previous research. Although not the primary client, impact on those known to a victim/client is important to address because it will inadvertently impact the victim, even if the victim’s direct needs are met. In other words, regardless of individual treatment, a client will continue to be impacted by their environment and potential problems within their support and peripheral networks. Thus, the results identify an additional population with clinical needs who may be less immediately identifiable, but who may also be utilizing resources to address the impact needs they have. The proposed index for assessing impact could include impact on significant or peripheral others so that clinicians can identify those persons, link their symptoms to the stalking, and further refer them for assessment and support. Wholistic support for a client who has been stalked that includes family and significant others will result in better care and support and consequently a better estimation and recording of the impact of stalking. It will also help with researcher triangulation to bring together seemingly unrelated phenomena of impact (particularly those seeking mental and physical health support).

## Conclusion

This study examined the impact of stalking in a national sample of help seeking victims who self-reported stalking impact. Results identified new forms of impact, including impact on those other than the victim. The results suggest the need for an impact reporting index to inform the implementation of trauma informed practice by professionals and the need to review stalking behavior diversity as an indicator of heightened impact. Stalking is a crime with severe and varied impact on victims which necessitates professional support that is informed by the trauma and prior research literature. Our study has hopefully elucidated that trauma-informed professional support, triangulation of available services to victims and their social circle, and a comprehensive impact index should be a pressing focus of future research and practice.
